# Modification and psychometric evaluation of the child perceptions questionnaire (CPQ_11–14_) in assessing oral health related quality of life among Lithuanian children

**DOI:** 10.1186/s12903-018-0701-5

**Published:** 2019-01-05

**Authors:** Aistė Kavaliauskienė, Antanas Šidlauskas, Apolinaras Zaborskis

**Affiliations:** 1 Faculty of Odontology, Clinic of Orthodontics, Lithuanian University of Health Sciences, Medical Academy, J.Luksos-Daumanto street, 6, LT-50106 Kaunas, Lithuania; 2 Faculty of Public Health, Health Research Institute and Department of Public Health, Lithuanian University of Health Sciences, Medical Academy, Tilzes street, 18, LT-47181 Kaunas, Lithuania

**Keywords:** Oral health, Quality of life, Child perceptions questionnaire, Psychometric analysis, Factorial validity, Children, Lithuania

## Abstract

**Background:**

Oral health related quality of life (OHRQoL) research among children and adolescents in Lithuania is just starting and no measures have been validated to date. Therefore, this study aimed to validate a Lithuanian version of the full (37 items) Child Perceptions Questionnaire (CPQ_11–14_) within a random sample of children aged 11 to 14.

**Methods:**

A cross-sectional survey among a randomly selected sample of schoolchildren (*N* = 307) aged 11 to14 was conducted. An anonymous questionnaire included the full CPQ_11–14_ and items on global life satisfaction, oral health and oral life quality self-rating. The questionnaire was translated into Lithuanian using translation guidelines. In addition, an item on the oral pain was modified identifying the pain location. Standard tests (Cronbach’s α, construct validity and discriminant validity), supplemented with both exploratory and confirmatory factor analyses, were employed for psychometric evaluation of the instrument. The questionnaire was also tested by comparison students’ and their parents’ (*N* = 255) responses about oral symptoms and functional limitations.

**Results:**

The modified Lithuanian version of CPQ_11–14_ revealed good internal consistency reliability (Cronbach’s alpha for the total scale was 0.88). The measure showed significant associations with perceived oral health status and oral well-being, as well as with global life satisfaction (*p* < 0.01). Discriminant validity of the instrument was approved by comparison of children’s groups defined by self-reported caries experience and malocclusion. Factor analysis revealed a complex structure with two or three factors in each of four domains of the CPQ_11–14_. Excellent or acceptable levels of indices of model fitting with the given data were obtained for oral symptoms, functional limitations and emotional well-being domains, but not for the social well-being domain. A significant association between child and parental responses was found (intraclass correlation coefficient was 0.56 and 0.43, correspondingly in domains of oral symptoms and functional limitations).

**Conclusion:**

The Lithuanian version of the CPQ_11–14_ (with a modified item that identifies location of oral pain) appears to be a valid instrument to be used in further studies for measuring OHRQoL among 11 to 14 year old children in Lithuania.

**Electronic supplementary material:**

The online version of this article (10.1186/s12903-018-0701-5) contains supplementary material, which is available to authorized users.

## Background

The last decades have seen an increasing importance in the literature of a concept that has come to be called oral health related quality of life (OHRQoL), which is applied in adult [1] as well as children and adolescents [2, 3] populations. Although there is no consensus on the definition of OHRQoL in children and adolescents, nor what aspects should be measured, it is generally accepted that OHRQoL is a multidimensional construct [4, 5]. It encompasses factors with four broad dimensions: the existence of discomfort or pain; functional factors; psychological factors; and social factors. Exhaustive systematic reviews [6, 7] have identified several validated instruments that currently exist to measure children’s OHRQoL: Child-Oral Impacts of Daily Performances index [8], Child Oral Health Impact Profile [9], Pediatric Oral Health-Related Quality of Life [10], and Child Perceptions Questionnaire (CPQ) [11].

The CPQ was, nevertheless, the first and most widely used inventory designed to assess the impact of oral conditions on quality of life in children [6, 7]. It was developed in 2002 by Jokovic et al. [11] as the CPQ_11–14_ for children aged 11 to 14 and was originally validated in children with caries, malocclusion and craniofacial anomalies. In terms of cognitive development, age specific versions of this tool have been produced [12], but the majority of studies used the original version CPQ_11–14_. The CPQ also has an analogous Parental Child Perceptions Questionnaire, which can be used as a proxy to CPQ [13]. The original item pool of the CPQ consists of 37 items, but the authors have also determined the psychometric properties of its shortened forms [14]. All variations of the questionnaire evaluate the impact of oral and orofacial conditions in children at symptomatic, functional, emotional and social levels. To date, the CPQ has been translated, validated and adapted to suit a number of languages and socio-cultural contexts demonstrating its applicability and perfect psychometric properties on numerous clinical and epidemiological occasions [6, 15–24].

Several methods have been employed in cross-cultural validations of the CPQ, however, the majority of such types of studies were realised without having carried out a validation process with factorial analysis. Factorial technique that includes exploratory factor analysis (EFA) and confirmatory factor analysis (CFA) is commonly used to inform the structure of the instrument (e.g. quality of life (QoL) measures) [25–27]. Such techniques have been employed in CPQ validation studies among Hong Kong adolescents [28, 29]. Findings from these studies indicated that the model using all 37 items fitted the data below the acceptable level. Thus, in order to validate and adapt the CPQ for any culture, it is rationale to analyse its dimensional structure.

OHRQoL research among children and adolescents in Lithuania is just starting and no measures have been validated to date. Given the positive CPQ properties and its high applicability for both clinical assessments and large-scale population studies, we have chosen this instrument for measure of OHRQoL in our research. It has also been considered that the original long form (37 items) of this instrument is more sensitive to changes in oral conditions rather than its short forms [14], hence, the original questionnaire was taken in focus. Each time a measurement scale is used in a new context or with a different population group, it is necessary to test its psychometric properties [30]. However, despite widespread use of the CPQ in many languages and cultures, it has never been adapted for use as a research tool in Lithuania. Therefore, the aim of this study was to validate a Lithuanian version of the full (37 items) CPQ_11–14_ within a random sample of children aged 11 to 14. The specific objectives were: 1) to translate the original CPQ_11–14_ into Lithuanian and to make modifications if needed; 2) to explore psychometric properties of the Lithuanian instrument version, and 3) to analyse its factorial structure.

## Methods

### Participants and data collection

The study followed a cross-sectional design and targeted adolescents aged 11 to 14 years. A number of 323 respondents was calculated to be sufficient for assessment of the prevalence of orthodontic anomalies in children, hypothesizing their prevalence to be 30% with 95% confidence interval from 25 to 35% [31]. The hypothesized prevalence of orthodontic anomalies was in agreement with our study in Lithuania among schoolchildren aged 11–15 years [32]. With regard to factorial analysis, this number of respondents is also sufficient, as the sample size should be at least 5 × *k*, where *k* is the number of items in factorial analysis (in our study *k* = 37) [33]. Then, accounting for anticipated non-response and participants who do not meet age requirements (11 to 14 years), the primarily sample size was increased in 50% (480 persons). The study sample was comprised of students from general education governmental schools in Lithuania. A list of schools and number of students by class was obtained from the education management information system of the Lithuanian Centre of Information Technologies in Education.

A two-stage cluster sampling method was used to draw a representative sample of students. In the first stage, 16 schools were randomly selected, ensuring equal presentation of urban and rural administrative regions. In the second stage, classes of students attending grades six to nine were chosen with a probability proportionate to the number of students in school. Thus, the primary sampling unit (cluster) was a class. In total, 25 classes were selected with estimated a required total number of students. Although the primary sampling unit was the class, a clustered sampling design effect was not accounted for either in the sample size calculation, nor in the analyses of data, as it was considered that students within the same class were similar to each other in oral health behavior, though they may not be as similar as in other health behavior patterns (e.g. smoking or bullying [34]).

School authorities were contacted by researchers and informed about all aspects of the study. Class tutors of selected classes were instructed about the process of carrying out the survey among students and their parents. They sent a description of the study, asking for written informed consent that their child be allowed to participate in the study to students’ parents. Of the 463 parents who initially received a request for written informed consent, positive answers were obtained from 393 (85%) of them. Those parents who gave consent were also asked to complete a questionnaire about their child’s oral health and well-being. The number of correctly completed parents’ questionnaires was 315 (68% of parents who were initially contacted). The students’ questionnaires were administrated in school classrooms. Eligible participants could freely choose to participate or not in the survey. Measures of anonymity and confidentiality were ensured. Respondents did not write their name in questionnaires, and upon completing the questions, they sealed the questionnaires in provided envelopes. A total of 381 students correctly completed the questionnaires, but 74 were excluded from the present analysis because of the students’ age criterion.

The final number of students, aged 11 to 14 years, whose questionnaires were used in the present study, was 307 (66% of initial sample size). Maintaining the same fieldwork methods, the data were collected during the 2013/2014 school year (*n* = 179) and in 2016/2017 school year (*n* = 128). The number of questionnaires completed both by the child and parents was 255. The gathering of questionnaires was ensured by using codes.

### Measures

#### CPQ instrument

The originally proposed CPQ_11–14_, adopted for children aged 11 to 14 years is a 37-item instrument consisting of four hypothesized health domains (subscales): (1) oral symptoms (OS, 6 items), (2) functional limitations (FL, 9 items), (3) emotional well-being (EWB, 9 items), and (4) social well-being (SWB, 13 items) [11]. The respondents were asked to indicate the frequency of a specified event in the past three months. Each question was asked to the respondents in the same way, “In the past three months, how often have you …(had/been + specified item) … because of your teeth/mouth?”. Answer options were: (0) ‘never’; (1) ‘once or twice’; (2) ‘sometimes’; (3) ‘often’; (4) ‘every or almost everyday’. The list of all of 37 CPQ items by health domains can be seen in Table 1.

**Table 1:** CPQ full questionnaire with 37 items and assessments of their impact on the domain reliability (*N* = 307)

Domain	Item code	Specified event^a^	Corrected item-total correlation^b^	Cronbach’s alpha if item deleted	Loadings in 1-factor solution
OS	O1	Pain in teeth, lips, jaws or mouth	0.316	0.626	0.497
	O2	Bleeding gums	0.311	0.634	0.493
	O3	Mouth sores	0.223	0.647	0.402
	O4	Bad breath	0.421	0.584	0.679
	O5	Food caught in or between teeth	0.546	0.525	0.784
	O6	Food stuck to roof of mouth	0.530	0.576	0.761
FL	F1	Breathing trough mouth	0.465	0.658	0.600
	F2	Taken longer than others to eat a meal	0.307	0.695	0.449
	F3	Trouble sleeping	0.425	0.665	0.553
	F4	Difficulty to bite or chew food like apples, corn on the cob or steak	0.382	0.674	0.538
	F5	Difficulty to open mouth wide	0.450	0.667	0.672
	F6	Difficulty to say any words	0.337	0.687	0.462
	F7	Difficulty to eat foods you would like to eat	0.467	0.657	0.660
	F8	Difficulty to drink with a straw	0.346	0.685	0.561
	F9	Difficulty to drink or eat hot or cold foods	0.300	0.689	0.468
EWB	E1	Irritable or frustrated	0.320	0.755	0.247
	E2	Unsure of himself	0.174	0.758	0.165
	E3	Shy or embarrassed	0.541	0.732	0.568
	E4	Concerned what other people think about you	0.666	0.711	0.868
	E5	Worried that is less attractive than other people	0.647	0.703	0.866
	E6	Upset	0.608	0.712	0.816
	E7	Nervous or afraid	0.376	0.746	0.342
	E8	Worried that is less healthy than other people	0.644	0.708	0.848
	E9	Worried that is different than other people	0.575	0.716	0.808
SWB	S1	Missed school	0.626	0.847	0.724
	S2	Hard time paying attention in school	0.601	0.848	0.668
	S3	Difficulty doing homework	0.543	0.852	0.607
	S4	Avoiding to speak or read out loud in class	0.562	0.851	0.657
	S5	Avoiding activities like sports, clubs, drama, music, school trips	0.605	0.849	0.700
	S6	Avoiding to talk to other children	0.625	0.847	0.718
	S7	Avoiding smiling or laughing when around other children	0.464	0.864	0.552
	S8	Difficulty playing a musical instrument such as a recorder, flute, clarinet, trumpet	0.402	0.860	0.464
	S9	Avoiding to spend time with other children	0.628	0.847	0.727
	S10	Argued with other children or family	0.546	0.852	0.633
	S11	Teased or called names by other children	0.336	0.863	0.406
	S12	Left out by other children	0.556	0.853	0.649
	S13	Asked questions by other children	0.574	0.850	0.642

*OS* oral symptoms, *FL* functional limitations, *EWB* emotional well-being, *SWB* social well-being

^a^Full wording of questions was “In the past 3 months, how often have you …(had/been + specified event) … because of your teeth/mouth?” and answer options were: (0) ‘never’; (1) ‘once/twice’; (2) ‘sometimes’; (3) ‘often’; (4) ‘every/almost every day’. ^b^ *p* < 0.001 for all values

The Lithuanian version the CPQ instrument conformed to concepts of the original version and was elaborated on using translation procedures (see below). Nevertheless, the content of the original item on the pain in teeth, lips, jaws or mouth (item O1) was discussed due to its complexity and wide scope of meaning. It was decided upon to change this item with five sub-items specifying a location of the pain (in teeth, lips, gums, oral mucosa and jaws or joints). A response option with the highest score that occurred throughout all these sub-items was considered as a response to the original item.

#### Rating of oral health and oral well-being

In order to validate the CPQ instrument, which was carried out in its developers’ study [11], the respondents were asked to rate their oral health and the extent to which it affected their well-being. For each of these dimensions, five sub-items were worded in the following way: a) “How you would describe health status of the following oral parts: - teeth; - lips; - gum; - oral mucosa; - jaws or joints?” and b) “Over the last three months, how much has your overall life been affected by the conditions of the following oral parts: - teeth; - lips; - gum; - oral mucosa; - jaws or joints?” The responses were scored in the following way: with regard to oral health rating: (0) ‘excellent’; (1) ‘very good’; (2) ‘good’; (3) ‘fair’, and (4) ‘poor’; with regard to well-being: (0) ‘not at all’; (1) ‘very little’; (2) ‘somewhat’ (3) ‘a lot’; and (4) ‘very much’. The sum score of all sub-items for each dimension ran from 0 to 20.

#### Global life satisfaction

The global life satisfaction measure was used as an additional tool in assessing construct validity of the CPQ. Children’s global life satisfaction was rated using the measurement technique from the HBSC study [34]. Children were asked to take a look at a picture of a ladder that had steps numbered from zero (“0”) at the bottom to ten (“10”) at the top, with an instruction to suppose the top of the ladder represents the best possible life, and the bottom of the ladder represents the worst possible life. They were asked to indicate the step of the ladder at which they would place their lives at present. The response was scored from zero to ten.

#### Family affluence

Family affluence is an important predictor of quality of life in young people [34], and therefore, it was decided to include it into the present study as a tool in assessing discriminant validity of the CPQ. It was measured by the Family Affluence Scale (FAS), which was specially developed for the HBSC study as a measure of social position [35]. The scale is simple and easy to answer even for children. The present FAS included four questions, including questions regarding car and home computers ownership, own bedroom occupancy and travelling on holidays. A composite FAS score was calculated for each respondent based on his or her responses to these four items, and then a three-point ordinal variable was composed for the present analysis, in which: score = 0–3 indicated low affluence; score = 4–5 indicated middle affluence, and score = 6–7) indicated high affluence.

#### Self-reported rating of experience with caries and malocclusion

Children were asked to answer: a) whether they have dental caries (tooth decay) or cavities that need to be treated, and b) if they have ever noticed that their teeth grew or were situated in an irregular way, or they have malocclusion. The answer categories for each question were: (1) ‘yes, I just noticed myself’; (2) ‘yes, this was confirmed by dentist’; or (3) ‘no’. In analyzing each question, the first two categories were combined. Thus, two sub-groups of respondents (correspondingly ‘not healthy’ and ‘healthy’) were selected separately for caries experience and malocclusion rating.

### Translation into Lithuanian

#### Forward translation into Lithuanian

The procedure of translation and national adaptation of the questionnaire followed guidelines proposed by Beaton et al. [36]. The principles of good practice proposed by International Society for Pharmacoeconomics and Outcomes Research were also taken into consideration [37]. The initial English version of the CPQ was taken from Shoroog Agou’s dissertation [38] and compared with the versions used in other validation studies [28]. It was first forward translated into Lithuanian by a co-author (AK) of this study, who is very familiar with the concepts included in the CPQ. Her mother-tongue language is Lithuanian and she is fluent in English. During this phase, the main focus was to achieve semantic, idiomatic, conceptual and scientific equivalence between the English and Lithuanian versions while adopting a vocabulary easily comprehensible for children. Then, the translated questionnaire was reviewed by all study co-authors. Ambiguities in translation were discussed with an invited language professional.

#### Back translation into English

The Lithuanian version of the CPQ was then blindly back-translated into the English language by a professional translator, fluent in English and unfamiliar with the concepts of the CPQ and original English version. The back-translated English questionnaire was compared to the original one, aiming to discern possible discrepancies and to solve any inconsistencies between the two versions. A multidisciplinary committee that comprised all the study co-authors, the back-translator and a school teacher, who had a postgraduate degree in English, discussed the differences between the initial English and Lithuanian versions of the questionnaire. A consolidated Lithuanian version was approved by consensus.

#### Pre-testing

Prior to the main study, a pilot test was carried out on a sample of students (*N* = 48) in one school. It was aimed to verify the level of understanding of the wording used and, where appropriate, to make any necessary changes. This so called ‘face validity’ test confirmed the feasibility of the methodology and showed that the young respondents had a high level of understanding of the questionnaire, whilst their suggestions enabled slight changes to be made to the wording, specifically regarding the questions about emotional and social well-being. Pre-testing of the question on pain in teeth, lips, jaws or mouth (item O1) that was modified by specifying a location of the pain demonstrated a high level of its understanding and appropriate distribution of answers by location of the pain. The final Lithuanian version of the CPQ is presented in Additional file 1.

### Statistical analysis

#### Descriptive statistics

The data were computerised and analysed using the SPSS statistical package supplemented with AMOS (version 21; IBM SPSS Inc., Chicago, IL, 2012). Missing responses to the CPQ items were substituted with the student’s mean score in a health domain if more than 2/3 of the domain items were completed; otherwise, the record was excluded from the analysis. The scores for each item were added together to obtain a sum score of each health domain, as well as of the total CPQ. Then, the sum scores were standardized to a percentage scale of 0–100% by dividing their value by the maximum sum score and multiplying by 100. Note that higher sum/percentage scores refer to worse OHRQoL.

The distributions of each item and the sum scores were examined. The sum scores of CPQ and its domains were found not to be normally distributed, thus, they were described using the median and the interquartile range (IQR). The null hypotheses that medians are the same across groups were therefore tested using median test. Upon the same reason, binary associations between variables were evaluated with non-parametric Spearman correlation coefficient. The cut-off level for statistical significance was set at 0.05.

#### Psychometric properties

A set of tests was used for examining psychometric properties of the CPQ [30, 33, 39]. The Cronbach’s alpha and intraclass correlation coefficient (ICC) average measure (one-way random effects) were used as a measure of internal consistency reliability of the total instrument and its domains. Values of ≥0.70 were considered acceptable [39]. Furthermore, other tests of internal reliability (inter-item and item-total correlations) were also investigated.

Construct validity of the instrument was tested using Spearman correlation coefficient to assess the association between the scores of total scale, as well as its domains and the respondents’ rating of their oral health, oral health related well-being and the global rating of life satisfaction. Discriminant validity was tested by comparing the medians of scores between groups (median test) defined by gender, age, social position, subjective caries experience and malocclusion traits.

A test-retest reliability test of the instrument was not employed; instead, we assessed agreement between children’s and their parents’ answers to the same questions of the OS and FL domains. The association between child and parental sum scores was assessed by Spearman correlation coefficient, and agreement between two groups of raters was evaluated by the ICC using two way mixed consistency method and the quadratic weighted kappa [39]. The quadratic weighted kappa was used due to high range of sum scores.

#### Exploratory factor analysis

Using the SPSS *Principal Component Factor Analysis* procedure, an EFA was performed on the set of items of each CPQ health domain. The suitability of the data for such analysis was tested using the Kaiser-Meyer-Olkin (KMO) measure of sampling adequacy, along with the Bartlett’s test of sphericity (KMO ≥ 0.5 and *p* < 0.001 show the adequacy of the data for use in the EFA). Initially, we explored 1-factor solution that ranks the items by their impact to the total variance of the domain. Then, the factors were extracted on the eigenvalues (> 1) with a *Promax* rotation. This solution helped to understand the interrelations among the items and to confirm the inherent structure of the instrument since factors were correlated. Factor loadings less than 0.4 indicate low item impact on the validity of the instrument [25, 33].

#### Confirmatory factor analysis

CFA [27, 33, 40, 41] was employed to establish factorial validity of the CPQ domains. The goodness of fit of the explored models was evaluated using multiple fit indices. Relative chi-square (χ^2^/df) and its *p*-value, comparative fit index (CFI), Tucker–Lewis index (TLI) and root mean square error of approximation (RMSEA) were taken into account. Relative chi-square is the chi-square ratio to degrees of freedom, and it is suggested that its value less than three or a non-significant *p*-value corresponds to an acceptable fit, however, the chi-square increases with sample size and model complexity and, therefore, this test was complemented by other tests [33, 42]. The values of CFI and TLI values close to 1 (≥.90) are commonly indicated as acceptable model fits [42]. An RMSEA value between 0.08 and 0.10 indicates an average fit, and a value below 0.08 and below 0.05 shows correspondingly a good and excellent fit [42]. We performed CFA on each of four domains (OS, FL, EWB and SWB). Initial models were based on the above EFA postulating that the domains should have as many latent variables as many common factors were established and that latent variables might be correlated. In order to achieve the optimum combination of simplicity and fit of the final models, the options of *Heuristic Specification Search* and *Modification Indices* in AMOS were employed [40].

## Results

### Sample characteristics

Self-reported data were collected from 307 children aged 11 to 14 years. The mean age of the children was 13.27 years (standard devition (SD) = 1.01), and 128 (41.7%) were boys. The respondents represented all social groups by family affluence levels: 32 (10.7%) of low, 105 (35.1%) of middle and 162 (54.2%) of high (8 missing cases) level; and by residence area: 221 (72.2%) of urban and 86 (27.8%) of rural residence.

### Measure modification

In our study, a single item on the pain in teeth, lips, jaws or mouth from the OS domain was substituted by a series of five sub-items identifying a location of oral pain. The distribution of respondents’ answers to these sub-items is presented in Table 2. Responses provided a variety of locations of oral pain, although pain in teeth seemed to be the most frequent complaint. The new item that combined responses to all sub-items showed that 67.3% of respondents have experienced pain in teeth, lips, jaws or mouth at least one or two times in the prior three months. In further analyses, this variable was used as the original item OS1 “Pain in teeth, lips, jaws or mouth”.

**Table 2:** Responses of the respondents to items on the oral pain location and distribution of the item that combined the pain in teeth/mount (*N* = 307)

Item		Never	One or two times	Sometimes	Often	Everyday or almost everyday
		*n* (%)	*n* (%)	*n* (%)	*n* (%)	*n* (%)
O1.1	Pain in teeth	156 (50.8)	111 (36.2)	37 (12.1)	2 (0.6)	1 (0.3)
O1.2	Pain in lips	243 (79.2)	43 (14.0)	13 (4.2)	6 (2.0)	2 (0.6)
O1.3	Pain in gum	243 (79.2)	54 (17.6)	8 (2.6)	1 (0.3)	1 (0.3)
O1.4	Pain in oral mucosa	276 (89.9)	20 (6.5)	10 (3.3)	1 (0.3)	0
O1.5	Pain in jaws or joints	266 (86.6)	35 (11.5)	5 (1.6)	0	1 (0.3)
O1	Pain in teeth, lips, jaws or mount	103 (33.6)	140 (45.6)	50 (16.3)	9 (2.9)	5 (1.6)

### Reliability analysis

The response rate to the items of the CPQ ranged from 97.1 to 100% with the highest rate of blanks (2.9%) in responses to item S10 “Argued with other children or your family because of your teeth or mouth” (SWB domain).

The impacts, that is the items scored from 1 (‘1 or 2 times’) to 4 (‘everyday or almost everyday’), were reported most frequently in the OS domain (“Pain in teeth, lips, jaws or mouth” – 67.3%; “Food stuck in or between teeth” – 59.6%; “Bleeding gums” – 56.8%; “Bad breath” – 47.9%) and in the EWB domain (“Worried that he/she is not as good looking as others” – 42.4%; “Worried that he/she is not as healthy as others” – 32.4%).

Descriptive statistics of the total CPQ and its health domains are presented in Table 3. Sum scores were found to be highly skewed and not normally distributed in all the health domains with a very noticeable floor effect, especially in the SWB domain. Out of the theoretical range of 0–100% of transformed scores, their mean (except OS domain) and median did not exceed 20%.

**Table 3:** Summary statistics of the CPQ and its domains (*N* = 307)

Domain / total CPQ	Mean	SD	Median	IQR	Skewness
OS	20.87	16.21	16.67	16.67	1.23
FL	7.75	11.65	3.70	11.11	2.10
EWB	12.56	18.86	7.41	18.52	2.66
SWB	4.02	9.94	0	2.56	4.54
Total CPQ	9.73	10.23	6.31	10.81	1.78

*SD* standard deviation, *IQR* interquartile range

Assessments of internal consistency reliability of the CPQ and its domains are displayed in Table 4. Cronbach’s alpha for the total CPQ was 0.875. For the domains, the coefficient ranged from 0.645 for OS domain to 0.862 for SWB domain, indicating from an acceptable to good internal consistency reliability. Similar assessments were found for the ICC.

**Table 4:** CPQ and its domains internal consistency reliability statistics ^a^ (*N* = 307)

Domain/total CPQ	Number of items	Cronbach’s alpha	ICC (95% CI)
OS	6	0.645	0.568 (0.488–0.639)
FL	9	0.701	0.691 (0.637–0.741)
EWB	9	0.759	0.755 (0.712–0.794)
SWB	13	0.862	0.859 (0.835–0.882)
Total CPQ	37	0.875	0.860 (0.836–0.881)

*ICC* intraclass correlation coefficient, *CI* confidence interval. ^a^ *p* < 0.001 for all values

All items of the OS and EWB subscales were found to be significantly inter-item correlated, while this was not achieved for the items of FL and SWB subscales (data not shown). The item-total correlations were significant at a 0.01 level for all domains, as well as for the total CPQ (see Table 1). These results, in combination with Cronbach’s alpha if item deleted and a 1-factor solution from the EFA, ranked the items by their impact to the total variance of the domain and indicate which items have the weakest impact within the domain and, consequently, could be removed from the corresponding domain. The items “Sores in mouth” (O3), “Irritable or frustrated” (E1), “Unsure of himself” (E2), “Difficulty playing a musical instrument such as a recorder, flute, clarinet, trumpet” (S8), and “Teased or called names by other children” (S11) are examples of such kind (see Table 1).

### Construct validity

Table 5 displays the correlation between scale sum scores and overall ratings of oral health and well-being, as well as with global life satisfaction. All domains and total CPQ were found to be significantly (*p* < 0.01) and positively correlated with oral health and oral well-being. The correlations between the global life satisfaction and the domains were all significant (a negative correlation value indicates that higher life satisfaction is related to lower rating of oral problems).

**Table 5:** Construct validity: Spearman correlation of the total CPQ and its domains with overall ratings of oral health, oral well-being and global life satisfaction (*N* = 307)

Domain/total CPQ	Spearman correlation^a^		
	Oral health	Oral well-being	Global life satisfaction
OS	0.328	0.475	− 0.259
FL	0.237	0.358	−0.172
EWB	0.312	0.382	−0.317
SWB	0.179	0.280	−0.171
Total CPQ	0.359	0.491	−0.327

^a^All correlations are significant at *p* < 0.01

### Discriminant validity

Discriminant validity of the instrument was tested by assessing CPQ scores by gender, family affluence, self-reported caries experience and malocclusion traits (Table 6). A significant gender difference in the EWB domain, as well as in the total CPQ revealed that there was poorer emotional well-being among girls. Across the groups of adolescents by family affluence level, a significant gradient within the family affluence groups was observed overall as well as in EWB and SWB domains. Adolescents who subjectively reported experience with caries in comparison with their ‘healthy’ contemporaries indicated significantly greater scores in the OS, FL and EWB domains but not in the SWB domain. In parallel with this, self-reported malocclusion also indicated a negative impact on the overall OHRQoL, but was significant within the OS and EWB domains only.

**Table 6:** Discriminant validity: Median (IQR) of the CPQ and its domains, by gender, family affluence, self-reported rating of caries experience and malocclusion

	*n*	Domain				Total CPQ
		OS	FL	EWB	SWB	
*Gender*						
boys	128	16.67 (16.7)	3.70 (7.4)	3.70 (14.8)	0 (2.6)	5.41 (9.2)
girls	179	16.67 (16.7)	3.70 (11.1)	7.40 (18.5)	0 (5.1)	6.31 (10.8)
*p*^a^		0.993	0.386	0.007	0.084	0.016
*Family affluence:*						
low	32	22.22 (22.2)	3.70 (21.3)	14.81 (29.6)	1.28 (10.3)	11.26 (16.2)
middle	105	22.22 (22.2)	3.70 (11.1)	7.41 (18.5)	0 (5.1)	7.21 (12.2)
high	162	16.67 (22.2)	3.70 (7.4)	3.70 (14.8)	0 (2.6)	4.51 (9.0)
*p*^a^		0.159	0.340	0.010	0.047	0.009
*Self-reported rating of caries experience:*						
'healthy’	207	16.7 (16.7)	3.7 (11.1)	3.7 (14.8)	0 (2.6)	5.4 (10.8)
'not healthy’	98	22.2 (22.2)	7.4 (14.8)	11.1 (25.9)	0 (5.1)	8.1 (13.1)
*p*^a^		< 0.001	0.007	0.013	0.356	< 0.001
*Self-reported rating of malocclusion:*						
'healthy’	136	16.7 (22.2)	3.7 (10.2)	3.7 (11.1)	0 (2.6)	5.4 (8.1)
'not healthy’	170	22.2 (22.2)	3.7 (14.8)	9.3 (25.9)	0 (5.1)	8.1 (12.6)
*p*^a^		0.009	0.119	< 0.001	0.076	< 0.001

^a^Median test, underlined values indicate a significant difference between medians in groups

### Agreement between child and parental reports

It was possible to compare 255 pairs of OS and FL as a sum score rated by children and their parents (Table 7). Positive significant correlations, which obtained value of a moderate level, were observed for sum scores of both domains. The moderate values of kappa and ICC also confirmed agreement between child and parental reports. These results suggest reliability of two subscales of the CPQ in respect of agreement between two different raters.

**Table 7:** Agreement between child and parental reports about oral symptoms (OS) and functional limitations (FL) (*N* = 255)

	OS	FL
Spearman correlation coefficient	0.419^**^	0.305^**^
Quadratic weighted kappa	0.400^***^	0.326^***^
Intraclass correlation coefficient (95% CI)	0.557^***^ (0.433–0.654)	0.429^***^ (0.269–0.553)

** *p* < 0.01; *** *p* < 0.001

### Results of the exploratory factor analysis

Table 8 presents the factor structure of each domain of the CPQ obtained from the EFA. The appropriateness of these factor models was evaluated by Bartlett’s test of sphericity (*p* < 0.001 for all four domains) and KMO measure of sampling adequacy (it ranged from 0.666 in FL domain to 0.862 in EWB domain). The analysis revealed a complex factor structure in all domains of the CPQ. The estimated loadings indicate that the domain of oral symptoms (OS) includes two factors that explained 38.4 and 16.6% of the total variance correspondingly. Factor 1 combined four items (“Bleeding gums” (O2), “Food caught in or between teeth” (O5), “Bad breath” (O4), and “Food stuck to roof of mouth” (O6)), while the factor 2 combined two items (“Mouth sores” (O3), and “Pain in teeth, lips, jaws or mouth” (O1)) (items are listed by loading weights). Analysis of the FL domain showed that at least two factors could be extracted, which explain 31.0 and 16.1% of the total variance correspondingly. Factor 1 combined six items on limitations of the eating function (F5, F8, F7, F9, F4, F2), and factor 2 combined breathing (F1), sleeping (F3) and speaking (F6) disorders. The items of the EWB domain were split explicitly into two factors that explained 45.2 and 20.5% of the total variance correspondingly. Factor 1 combined five items (E4, E5, E9, E8, E6) that describe adolescent emotions, while factor 2 combined the remaining four items (E1, E2, E7,E3) concerned with adolescent personality. Items of the SWB domain showed a three factor structure, explaining 40.2, 14.2 and 8.5% of the total variance by the corresponding factors. Factor 1 combined six items (S10, S7, S12, S9, S13, S6) specifying the adolescent’s isolation from his/her peers; factor 2 combined five items (S2, S3, S8, S1, S4) on adolescent difficulties in school work and out-of-school activity; and factor three combined two items (S11, S5) that measured disorders in adolescent relations with other children due to his/her oral problems.

**Table 8:** Factor loadings from the Exploratory Factor Analysis, by the CPQ domains^a^ (*N* = 307)

Domain	Item	Factor 1	Factor 2	Factor 3
OS (KMO = 0.746)	O2 Bleeding gums	0.721	−0.343	
	O5 Food caught in or between teeth	0.712	0.187	
	O4 Bad breath	0.705	0.013	
	O6 Food stuck to roof of mouth	0.657	0.239	
	O3 Mouth sores	−0.129	0.927	
	O1 Pain in teeth, lips, jaws or mouth	0.290	0.474	
	Total variance explained (%)	38.4	16.6	
FL (KMO = 0.666)	F5 Difficult to open mouth wide	0.843	−0.070	
	F8 Difficult to drink with a straw	0.805	−0.177	
	F7 Difficult to eat foods you would like to eat	0.601	0.198	
	F9 Difficult to drink or eat hot or cold foods	0.528	0.021	
	F4 Difficult to bite or chew food like apples, corn on the cob or steak	0.400	0.267	
	F2 Taken longer than others to eat a meal	0.291	0.273	
	F1 Breathed trough mouth	−0.002	0.824	
	F3 Trouble sleeping	−0.046	0.812	
	F6 Difficult to say any words	0.005	0.626	
	Total variance explained (%)	31.0	16.1	
EWB (KMO = 0.862)	E4 Concerned what other people think about you	0.887	−0.004	
	E5 Worried that is less attractive than other people	0.884	−0.002	
	E9 Worried that is different than other people	0.862	−0.091	
	E8 Worried that is less healthy then other people	0.852	0.032	
	E6 Upset	0.838	−0.015	
	E1 Irritable or frustrated	−0.077	0.784	
	E2 Unsure of himself	−0.153	0.764	
	E7 Nervous or afraid	0.047	0.720	
	E3 Shy or embarrassed	0.345	0.557	
	Total variance explained (%)	45.2	20.5	
SWB (KMO = 0.788)	S10 Argued with other children or family	0.862	−0.173	0.059
	S7 Avoiding smiling or laughing when around other children	0.772	0.029	−0.180
	S12 Left out by other children	0.754	−0.075	0.103
	S9 Avoiding to spend time with other children	0.693	0.188	−0.009
	S13 Asked questions by other children	0.603	0.040	0.147
	S6 Avoiding to talk to other children	0.484	0.029	0.417
	S2 Hard time paying attention in school	0.000	0.863	−0.016
	S3 Difficulty doing homework	−0.184	0.863	0.123
	S8 Difficulty playing a musical instrument such as a recorder, flute, clarinet, trumpet	0.050	0.767	−0.266
	S1 Missed school	0.375	0.667	−0.172
	S4 Avoiding to speak or read out loud in class	−0.168	0.590	0.489
	S11 Teased or called names by other children	0.006	−0.252	0.874
	S5 Avoiding activities like sports, clubs, drama, music, school trips	0.140	0.180	0.643
	Total variance explained (%)	40.2	14.2	8.5

^a^Extraction Method: Principal Component Analysis on eigenvalue > 1. Rotation Method: Promax with Kaiser Normalization. The underlined terms indicate the main loadings for corresponding factors. KMO: Kaiser-Meyer-Olkin measure

A complex factor structure of the CPQ domains was also seen from noticeable loadings of several items (F2, F4, E3, S4, S6); therefore, they might be attributed to more than one specified factor. Further, the dimensionality of the CPQ domains was assessed employing CFA.

### Results of the confirmatory factor analysis

We performed CFA on each of four domains (OS, FL, EWB and SWB). Table 9 reports the goodness-of-fit statistics for the final models of each domain.

**Table 9:** Model fit estimations in the Confirmatory Factor Analysis, by the CPQ domains (*N* = 307)

Domain	χ^2^	df	χ^2^ / df	p-value	CFI	TLI	RMSEA (90% CI)
OS	12.1	8	1.511	0.147	0.977	0.956	0.041 (0.000–0.085)
FL	67.0	22	3.048	< 0.001	0.911	0.854	0.082 (0.060–0.104)
EWB	41.3	24	1.722	0.015	0.986	0.980	0.049 (0.021–0.073)
SWB	347.3	57	6.093	< 0.001	0.852	0.797	0.129 (0.116–0.142)

*df* degrees of freedom, *CFI* comparative fit index, *TLI* Tucker–Lewis index, *RMSEA* root mean square error of approximation, *CI* confidence limits

**Fig. 1:**
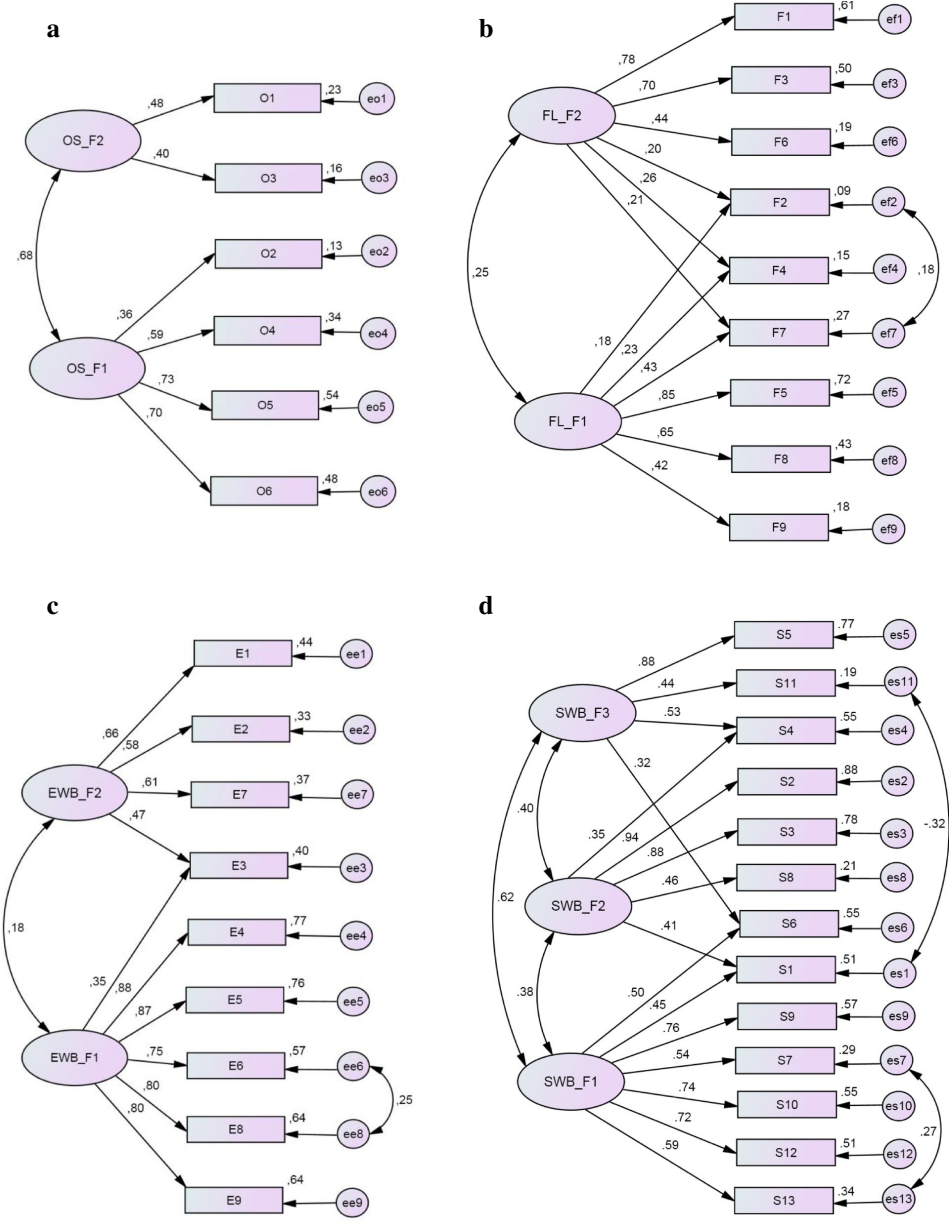
Path diagrams with standardized estimations of the final CFA models: **a**) oral symptoms (OS), **b**) functional limitations (FL), **c**) emotional well-being (EWB), **d**) social well-being (SWB)

Among four domains, the OS domain model had the best fit estimations, unless its internal consistency reliability (Cronbach’s alpha) was the lowest. Its goodness-of-fit indices showed excellent model fitting to the given data including relatively great *p*-value, which is not uncommon for such a sample size. As presented in Fig. 1a, the model includes two latent variables (factors). In accordance with the EFA solution, the items “Bleeding gums” (O2), “Food caught in or between teeth” (O5), “Bad breath” (O4) and “Food stuck to roof of mouth” (O6) went to factor one and the items “Pain in teeth, lips, jaws or mouth” (O1) and “Mouth sores” (O3) went to factor two. The item “Pain in teeth, lips, jaws or mouth” (O1) that was modified in our study had a significant positive impact on the factor two. Both factors were significantly correlated (*r* = 0.68, *p* < 0.001).

The model of the FL domain had acceptable fitting to the concerned data. It was in accordance with the two factors structure that was revealed in the EFA. Results shown in Fig. 1b illustrate a positive association between selected factors (*r* = 0.25, *p* < 0.001), unless they reflect different aspects of life quality related to limitations of oral functions. Both EFA and CFA confirmed low effect of the functional limitation “Taken longer than others to eat a meal” (F2).

The model of the EWB domain had excellent fitting indices and confirmed a two factor structure, as well as being revealed in the EFA (Fig. 1c). There was a positive correlation between factors (*r* = 0.33, *p* < 0.001). The item “Shy and embarrassed” (E3) provided unspecified position in factorial structure.

The model of the SWB, which included three latent variables, was checked. As a result, this model had a complex structure and was not well-fitted to the research data (Fig. 1d).

## Discussion

The CPQ_11–14_ inventory has long been acknowledged as a valid tool in OHRQoL research worldwide [15–22]. It has also been found to be valid in children with dental caries, malocclusion and craniofacial anomalies [6, 43]. Therefore, the present study aimed to validate a Lithuanian version of the CPQ and explore its psychometric properties in a sample of school children from national schools. Considering this point, our study is innovative in OHRQoL research in Lithuania, as past studies indicated that dental caries and orthodontic anomalies are widely spread among children in different age groups in Lithuania [32, 44].

The first specific objective of our research was to elaborate a Lithuanian version of the CPQ. We fulfilled guidelines translating the original questionnaire into Lithuanian, including back translation [36, 37]. In order to make the questionnaire more acceptable to the Lithuanian children, several changes were incorporated in the questionnaire. For instance, when the questionnaire was piloted, most of the children could not recall that they need to choose option ‘not at all’ if the health complaint was due to reasons other than those related to their oral health. In order to avoid this confusion, wording of several health complaints was appended with the phrase ‘because of your teeth or mouth’.

In the Lithuanian version of the CPQ the only one modification was done which is related with the first item of the OS domain “Pain in your teeth, lips, jaws or mouth” (O1). In our opinion, this item is very general, while a specification of the pain location could be important for further analysis of the CPQ properties. For instance, it is important to distinguish well-being between children with dental caries, malocclusion and craniofacial anomalies [11]. This may also reflect the difficulties children may have with the concept of ‘oral health’, because of that they may be referring oral health complains to gingival health and caries status rather than malocclusion [45]. Participants of the pilot test also commented that a group of questions with specification of the pain location was easy to answer. Moreover, this item modification could not restrict the properties of the originally designed CPQ as the value of original item could be restored using mostly expressed paint. Unfortunately, a discussion of whether this change could be meaningful was not included in any of validation studies of the CPQ.

A wide range of statistical tools have been used in previous studies and their results for validity and reliability of the original instrument and its versions translated into other languages were rated positively in all studies [6]. The findings of the second specific objective of our study also confirmed high internal consistency of the original CPQ with national data. Cronbach α for the total CPQ was 0.875, which is in accordance with that reported by the developers of the CPQ in their validation study [11]. In general, the internal consistency of our survey was in any case as high as that reported by other CPQ validation studies [15–24]. However, Cronbach α of the OS domain (the domain which was modified in our study) was below an acceptable level of 0.70. Its value (0.645) was lower than that found from the Brazilian version (0.75) [46] and the Italian version (0.90) [20]. However, this value was as low as that obtained in many other studies, e.g. from the Australian version (0.68) [24], the Canadian version (0.64) [11], the Korean version (0,64) [21], the Indian version (0.629) [22] and the UK version (0.59) [23]. There may be several reasons for explaining the low Cronbach α of the OS domain. Since the value of Cronbach α depends on the number of items that make up the scale [33, 39], the lowest its value can be explained by the fact that OS domain contains the smallest number of items. Moreover, the OS domain describes the variety of oral symptoms that may be not so much associated, so there is no need to expect a high value of internal consistency. On the other hand, our study found other good parameters (*p*-value > 0.05, CFI > 0.95; TLI close to 1; and RMSEA< 0.08) which indicate that the model of associations between items in the OS domain presented a good fit to real data. These findings have confirmed good validity of the CPQ including its OS domain.

Similarly to other studies, we analysed construct validity of the instrument by assessing correlation between sum scores of the scale and overall ratings of oral health and well-being. In contrast with other studies, we estimated additionally the relationship of sum scores with the child’s global life satisfaction that is essential dimension of young people well-being [34, 47]. All estimations of correlation were significant that indicates adequate construct validity of the Lithuanian CPQ version.

To confirm discriminant validity, the distributions of sum scores of the instrument between the sub-groups of respondents were compared. Girls comparing with boys demonstrated significantly greater sum scores of the total CPQ. With regard to health domains, a significant difference was found for EWB domain only. These findings are in accordance with conclusions reported by several other authors [20, 48]. Difference between genders may be attributed to the poorer girls’ perception life satisfaction [34, 49], as well as of OHRQoL [48, 50]. Olivieri et al. [20] in validation study of the CPQ Italian version and Simoes et al. [51] in their study of Brazilian schoolchildren described a significant gradient within the social classes overall as well as in the emotional and social well-being domains. Findings from our study confirmed conclusions of Italian and Brazilian studies. There are suggestions in the literature that children from high-income families usually present better oral hygiene habits and have more access to prevention and dental treatment; hence, these conditions may reflect in a better OHRQoL [51, 52]. These findings confirm the need to consider family wealth when studying OHRQoL and planning oral health strategies in children [50].

Distribution of the CPQ scores identified significant differences between ‘healthy’ and ‘not healthy’ sub-groups defined on the basis of subjective rating of caries experience and malocclusion. We found that children with self-reported caries experience, comparing with those who were aware not having caries in their teeth, reported significantly greater scores in all health domains. In literature, the evidence that CPQ is associated with dental caries in the general population is not still clarified yet as there have been conflicting findings from the validation studies with some findings confirming such association [15–17, 21] and others not [18–20, 22, 23, 46]. Concerning subjective rating of malocclusion, we found that malocclusion as well as caries experience has a significant negative impact on the OHRQoL of children in terms of the CPQ scores, except SWB health domain. This effect was mostly noticeable for OS and EWB domains. Systematic reviews confirm that there is an association between malocclusion/orthodontic treatment need and poor OHRQoL in children seeking orthodontic treatment [43, 53–55]. It was concluded that malocclusion has a considerable negative impact on psychosocial functioning of both children and their families [56]. Therefore, it has been suggested that severe malocclusions have an impact on OHRQoL predominantly in the emotional and social dimensions [43, 53, 54] or, in contrast with our findings, in social dimension only [45].

In the present study, test-retest reliability of the CPQ instrument was not assessed due to organizational and logical reasons. With regard to organizational reasons, a retest appeared problematic as organizing another survey at several schools participating in our study would have a complex endeavour. With the respect to logical reasons, a retest of the same students was replaced with an alternative analysis that included comparison of children’s and their parents’ answers to the same questions of the OS and FL sub-scales. Such comparison was not performed for the EWB and SWB sub-scales, because some parents may have limited knowledge about their children’s OHRQoL, particularly the impact on social and emotional well-being [57]. As in other similar studies in this field [57–59], findings of the present study confirmed an agreement between child and parental reports suggesting on reliability of the CPQ in respect of its repeatability by two different raters.

Finally, the third specific objective of our study sought to explore the factorial structure of the Lithuanian CPQ. This technique allows for a more detailed assessment of the questionnaire validity [30]. In the literature, the hypothesized factor structure of the CPQ in terms how well the items reflect their corresponding health domains hasn’t been set out so widely as in researches of other instruments [26, 60]. To our knowledge, a CFA has been applied only by Lau et al. (2009) in the CPQ validation study for children in Hong Kong [28, 29]. Based on the five supplementary goodness-of-fit indices, the authors concluded that full CPQ model with four first-order factors fit the data below acceptable level. Among the four health domains, only the OS and FL fitted the data adequately but not for the domains EWB and SWB. We adopted factorial analysis, including both EFA and CFA, into validation of a Lithuanian version of the CPQ. The EFA revealed a non-homogenous structure of each health domain, which consisted of two or three dimensions. The CFA tested the structure of each health domain. Excellent or acceptable characteristics of the goodness-of-fit for data of the national sample of children were found for three of four domains: OS, FL and EWB. The item “Pain in teeth, lips, jaws or mouth” (O1) had a significant positive loading in the OS structure, which demonstrates the success of its modification in our study. The SWB domain fitted the data as poorly as for children in Hong Kong [28], consequently, this domain may be considered as an object of future research.

### Strengths of the study

This study analysed data that were collected in cross-sectional population survey of young people samples but not within samples of patients attending dental treatment as in several studies [11, 45, 61, 62]. The children completed their questionnaires at school anonymously without any influence of their parents‘opinion, thus, children could express their own feelings towards their QoL that is an important condition comparing children’s and their parents responses. Employment of a CFA in testing of the CPQ instrument reliability that is unusual by most of the previous cross-cultural validation studies of CPQ_11–14_ also can to be considered as a novelty and strength of the study.

### Limitations

There are several limitations in the current study. First and foremost, in the present study the CPQ sum scores were compared with the self-reported caries experience and malocclusion traits/orthodontic treatment need but not with clinical dental examination outcomes. The literature also shows that several studies did not reveal any effect of caries and malocclusion on the OHRQoL [18–20, 22, 23, 46]. Moreover, although our modification in the studied instrument helped to identify the kind of oral pain but the discriminant validation on the instrument was limited to caries and malocclusion. We believe that these comparisons are worth of greater attention, so their analysis will be an objective for another study. Next, given the aim of the present study, we worked on the “long form” (37 items) of the original CPQ_11–14_ together with other scales, including such as eating behaviour and self-esteem. Practical guides to develop measurement scales show that an increase of number of items in the questionnaire may affect respondent’s accuracy, especially for children, providing inaccurate answers, which may, consequently, reduce reliability of the tested scale [30]. Moreover, we added five sub-items for the first OS item, increasing the number of items in the questionnaire. We believe that this type of change would be better applied to the short-form CPQ, and further research should confirm this. Another important consideration is the age limitation (from 11 to 14 years old) of children to whom the instrument is addressed. Jokovic et al. [11] discussed the role of child’s cognitive abilities on self-report health status and suggested to be used age-specific questionnaires for children younger than 11-year-old. Therefore, we raise the hypothesis that the CPQ_11–14_ measure in older adolescent group (e.g. aged 16–18 years) is more reliable as it is in 11–14-year-olds, however, to date we haven’t found any studies confirming this hypothesis [63]. Finally, in the present study, test-retest reliability of the CPQ instrument was replaced with an alternative analysis that included comparison of children’s and their parents’ responses to the same questions of the OS and FL domains. This approach is not free from limitations, especially in relation to its accuracy because children and parents may not share the same views about illness and health [11].

## Conclusions

The translated Lithuanian version of the CPQ_11–14_ with a modified item on the oral pain by identification its location demonstrated good internal consistency and construct and discriminant validity and appears to be a valid instrument to be used in further studies for measuring OHRQoL in Lithuanian children aged 11 to 14 years. However, employment of the factorial analysis revealed several weaknesses in dimensional structure of the social well-being domain, thus, a continuous psychometric analysis of the utilized instrument is recommended.

## Additional file

Additional file 1: Lithuanian version of the CPQ. (DOCX 44 kb)

## Abbreviations

CI: Confidence interval
CFA: Confirmatory factor analysis
CFI: Comparative fit index
CPQ: Child perceptions questionnaire
EFA: Explanatory factor analysis
EWB: Emotional well-being
FAS: Family affluence scale
FL: Functional limitations
HBSC: Health Behaviour in School-aged Children, a World Health Organization cross-national study
IQR: Interquartile range
KMO: Kaiser-Meyer-Olkin
N: Number of subjects/respondents/cases
OHRQoL: Oral health related quality of life
OS: Oral symptoms
QoL: Quality of life
RMSEA: Root mean square error of approximation
SWB: Social well-being
TLI: Tucker-Lewis index
