# Supplementary material for: Modification and psychometric evaluation of the child perceptions questionnaire (CPQ11–14) in assessing oral health related quality of life among Lithuanian children
Source: BMC Oral Health. 2019 Jan 5;19:1. doi: 10.1186/s12903-018-0701-5 (PMC6320629; doi:10.1186/s12903-018-0701-5)
Supplement: Supplementary file 1 — Lithuanian version of the CPQ. (DOCX 44 kb) [file 12903_2018_701_MOESM1_ESM.docx]

**ANKETA VAIKUI**

**CPQ_11-14_**

**Klausimai skirti CPQ_11-14_ psichometrinėms savybėms tirti**

| **Pasitenkinimas gyvenimu** | | | | |
| --- | --- | --- | --- | --- |
| Paveikslėlyje nupieštos kopėčios.  Kopėčių viršuje yra **10 –** labiausiai patenkintas savo gyvenimu; apačioje **0** – mažiausiai patenkintas savo gyvenimu.  **Kurioje vietoje ant kopėčių dabar Tu jautiesi stovintis?**  Pažymėk langelį, kuris geriausiai atspindi Tavo padėtį. |  |  | **10** | Labiausiai patenkintas gyvenimu |
|  |  |  | **9** |  |
|  |  |  | **8** |  |
|  |  |  | **7** |  |
|  |  |  | **6** |  |
|  |  |  | **5** |  |
|  |  |  | **4** |  |
|  |  |  | **3** |  |
|  |  |  | **2** |  |
|  |  |  | **1** |  |
|  |  |  | **0** | Mažiausiai patenkintas gyvenimu |

| Kaip apibūdintum savo burnos organų sveikatą? | | | | | | |  |
| --- | --- | --- | --- | --- | --- | --- | --- |
|  | | 1 | 2 | 3 | 4 | 5 | |
| ***Pažymėk po vieną langelį kiekvienoje eilutėje:*** | | *Labai*  *gera* | *Gera* | *Viduti-niška* | *Bloga* | *Labai bloga* | |
| 1 | Dantų |  |  |  |  |  | |
| 2 | Lūpų |  |  |  |  |  | |
| 3 | Dantenų |  |  |  |  |  | |
| 4 | Burnos gleivinės |  |  |  |  |  | |
| 5 | Žandikaulių ir jų sąnarių |  |  |  |  |  | |

| Ar per pastaruosius 3 mėnesius kaip nors Tave vargino burnos organų sveikatos sutrikimai? | | | | | | |
| --- | --- | --- | --- | --- | --- | --- |
|  | | 1 | 2 | 3 | 4 | 5 |
| ***Pažymėk po vieną langelį kiekvienoje eilutėje:*** | | *Visai nevargino* | *Truputį vargino* | *Kažkiek vargino* | *Vargino* | *Labai vargino* |
| 1 | Dantų |  |  |  |  |  |
| 2 | Lūpų |  |  |  |  |  |
| 3 | Dantenų |  |  |  |  |  |
| 4 | Burnos gleivinės |  |  |  |  |  |
| 5 | Žandikaulių ir jų sąnarių |  |  |  |  |  |

**CPQ_11-14_**

**Klausimai apie burnos sveikatos sutrikimus**

| Ar per pastaruosius 3 mėnesius jautei šių burnos organų skausmą? | | | | | | |  |
| --- | --- | --- | --- | --- | --- | --- | --- |
|  | | 1 | 2 | 3 | 4 | 5 | |
| ***Pažymėk po vieną langelį kiekvienoje eilutėje:*** | | *Niekada* | *Vieną ar du kartus* | *Kartais* | *Dažnai* | *Beveik kas dieną* | |
| O1.1 | Dantų |  |  |  |  |  | |
| O1.2 | Lūpų |  |  |  |  |  | |
| O1.3 | Dantenų |  |  |  |  |  | |
| O1.4 | Burnos gleivinės |  |  |  |  |  | |
| O1.5 | Žandikaulių ir jų sąnarių |  |  |  |  |  | |

| Ar per pastaruosius 3 mėnesius patyrei ir tokių burnos organų sutrikimų? | | | | | | |
| --- | --- | --- | --- | --- | --- | --- |
|  | | 1 | 2 | 3 | 4 | 5 |
| ***Pažymėk po vieną langelį kiekvienoje eilutėje:*** | | *Niekada* | *Vieną ar du kartus* | *Kartais* | *Dažnai* | *Beveik kas dieną* |
| O2 | Valant dantis kraujavo dantenos |  |  |  |  |  |
| O3 | Buvo opelių ar žaizdų burnos gleivinėje |  |  |  |  |  |
| O4 | Iš burnos sklido blogas kvapas |  |  |  |  |  |
| O5 | Lindo ir užsilaikė maistas tarp dantų |  |  |  |  |  |
| O6 | Užsilaikė maistas burnos ertmės viršuje (gomuryje) |  |  |  |  |  |

**Klausimai apie apribojimus, kuriuos patyrei dėl burnos sveikatos**

| Ar per pastaruosius 3 mėnesius dėl dantų arba burnos būklės Tau nutiko, kad Tu...?  *Jei tai nebuvo susiję su dantų arba burnos būkle, pažymėk atsakymą „Niekada“* | | | | | | |
| --- | --- | --- | --- | --- | --- | --- |
|  | | 1 | 2 | 3 | 4 | 5 |
| ***Pažymėk po vieną langelį kiekvienoje eilutėje:*** | | *Niekada* | *Vieną ar du kartus* | *Kartais* | *Dažnai* | *Beveik kas dieną* |
| F1 | Kvėpavai per burną |  |  |  |  |  |
| F2 | Ilgiau nei kiti valgei maistą |  |  |  |  |  |
| F3 | Blogai miegojai |  |  |  |  |  |

| Ar per pastaruosius 3 mėnesius dėl dantų arba burnos būklės Tu...? | | | | | | |
| --- | --- | --- | --- | --- | --- | --- |
|  | | 1 | 2 | 3 | 4 | 5 |
| ***Pažymėk po vieną langelį kiekvienoje eilutėje*** | | *Niekada* | *Vieną ar du kartus* | *Kartais* | *Dažnai* | *Beveik kas dieną* |
| F4 | Sunkiai atkandai ir kramtei kietą maistą, pvz., obuolį, mėsą |  |  |  |  |  |
| F5 | Negalėjai plačiai išsižioti |  |  |  |  |  |
| F6 | Negalėjai aiškiai tarti žodžius |  |  |  |  |  |
| F7 | Negalėjai valgyti to maisto, kurį Tu norėjai valgyti |  |  |  |  |  |
| F8 | Negalėjai gerti per šiaudelį |  |  |  |  |  |
| F9 | Negalėjai gerti arba valgyti nieko karšto arba šalto |  |  |  |  |  |

**Klausimai apie Tavo jausmus ir emocijas, kilusius dėl burnos sveikatos**

| Ar per pastaruosius 3 mėnesius dėl dantų arba burnos būklės Tu ...?  *Jei tai nebuvo susiję su dantų arba burnos būkle, pažymėk atsakymą „Niekada“* | | | | | | |
| --- | --- | --- | --- | --- | --- | --- |
|  | | 1 | 2 | 3 | 4 | 5 |
| ***Pažymėk po vieną langelį kiekvienoje eilutėje:*** | | *Niekada* | *Vieną ar du kartus* | *Kartais* | *Dažnai* | *Beveik kas dieną* |
| E1 | Verkei, greitai susijaudindavai |  |  |  |  |  |
| E2 | Trūko ryžto, pasitikėjimo savimi |  |  |  |  |  |
| E3 | Buvai drovus, jautei gėdą |  |  |  |  |  |
| E4 | Jaudinaisi, kad kiti blogai galvoja apie Tavo dantis arba burną |  |  |  |  |  |
| E5 | Jaudinaisi, kad Tavo dantys arba burna neatrodo taip gražiai kaip kitų |  |  |  |  |  |
| E6 | Jauteisi nusiminęs dėl savo dantų arba burnos |  |  |  |  |  |
| E7 | Buvai nervingas, irzlus, piktas |  |  |  |  |  |
| E8 | Jaudinaisi, kad Tavo dantys arba burna nėra tokie sveiki kaip kitų |  |  |  |  |  |
| E9 | Jaudinaisi, kad Tu išsiskiri iš kitų dėl savo dantų arba burnos išvaizdos |  |  |  |  |  |

**Klausimai apie mokyklą, draugus ir kitą veiklą**

| Ar per pastaruosius 3 mėnesius dėl dantų arba burnos būklės Tu ...?  *Jei tai nebuvo susiję su dantimis arba burna, pažymėk atsakymą „Niekada“* | | | | | | |  |
| --- | --- | --- | --- | --- | --- | --- | --- |
|  | | 1 | 2 | 3 | 4 | 5 | |
| ***Pažymėk po vieną langelį kiekvienoje eilutėje:*** | | *Niekada* | *Vieną ar du kartus* | *Kartais* | *Dažnai* | *Beveik kas dieną* | |
| S1 | Vengei lankyti mokyklą |  |  |  |  |  | |
| S2 | Negalėjai susikaupti pamokų metu |  |  |  |  |  | |
| S3 | Negalėjai susikaupti ruošiant namų darbus |  |  |  |  |  | |
| S4 | Klasėje vengei garsiai kalbėti arba skaityti |  |  |  |  |  | |
| S5 | Vengei lankyti sporto, choro ar kitus būrelius, vakarones ar iškylas mokykloje |  |  |  |  |  | |
| S6 | Vengei kalbėtis su draugais |  |  |  |  |  | |
| S7 | Vengei šypsotis ar juoktis, kai aplink buvo draugų |  |  |  |  |  | |
| S8 | Buvo sunku groti pučiamaisiais muzikos instrumen-tais (jei groji) |  |  |  |  |  | |
| S9 | Apskritai, vengei būti su draugais |  |  |  |  |  | |

Tęsti toliau ...

|  | | 1 | 2 | 3 | 4 | 5 |
| --- | --- | --- | --- | --- | --- | --- |
| ***Pažymėk po vieną langelį kiekvienoje eilutėje:*** | | *Niekada* | *Vieną du kartus* | *Kartais* | *Dažnai* | *Beveik kas dieną* |
| S10 | Kivirčijaisi su draugais ir namiškiais dėl savo dantų išvaizdos |  |  |  |  |  |
| S11 | Kiti vaikai iš Tavęs šaipėsi, Tave pravardžiavo |  |  |  |  |  |
| S12 | Kiti vaikai Tavęs vengė, nuo Tavęs šalinosi |  |  |  |  |  |
| S13 | Kiti vaikai klausinėjo, kas nutiko Tavo dantims arba burnai |  |  |  |  |  |
